# *In vivo* Exploration of the Connectivity between the Subthalamic Nucleus and the Globus Pallidus in the Human Brain Using Multi-Fiber Tractography

**DOI:** 10.3389/fnana.2016.00119

**Published:** 2017-01-19

**Authors:** Sonia Pujol, Ryan Cabeen, Sophie B. Sébille, Jérôme Yelnik, Chantal François, Sara Fernandez Vidal, Carine Karachi, Yulong Zhao, G. Rees Cosgrove, Pierre Jannin, Ron Kikinis, Eric Bardinet

**Affiliations:** ^1^Surgical Planning Laboratory, Department of Radiology, Brigham and Women’s Hospital, Harvard Medical School, BostonMA, USA; ^2^Department of Computer Science, Brown University, ProvidenceRI, USA; ^3^Institut du Cerveau et de la Moëlle Epinière, INSERM U 1127, CNRS UMR 7225, Sorbonne Universités, University of Paris 06, UMR S 1127Paris, France; ^4^Centre de Neuro-Imagerie de Recherche, Institut du Cerveau et de la Moëlle EpinièreParis, France; ^5^Department of Neurosurgery, Pitié-Salpêtrière HospitalParis, France; ^6^LTSI, Inserm UMR 1099 – Université de RennesRennes, France; ^7^Department of Neurosurgery, Brigham and Women’s Hospital, Harvard Medical School, BostonMA, USA

**Keywords:** diffusion MRI, subthalamic nucleus, globus pallidus, multi-fiber tractography, deep brain stimulation, human neuroanatomy

## Abstract

The basal ganglia is part of a complex system of neuronal circuits that play a key role in the integration and execution of motor, cognitive and emotional function in the human brain. Parkinson’s disease is a progressive neurological disorder of the motor circuit characterized by tremor, rigidity, and slowness of movement. Deep brain stimulation (DBS) of the subthalamic nucleus and the globus pallidus pars interna provides an efficient treatment to reduce symptoms and levodopa-induced side effects in Parkinson’s disease patients. While the underlying mechanism of action of DBS is still unknown, the potential modulation of white matter tracts connecting the surgical targets has become an active area of research. With the introduction of advanced diffusion MRI acquisition sequences and sophisticated post-processing techniques, the architecture of the human brain white matter can be explored *in vivo*. The goal of this study is to investigate the white matter connectivity between the subthalamic nucleus and the globus pallidus. Two multi-fiber tractography methods were used to reconstruct pallido-subthalamic, subthalamo-pallidal and pyramidal fibers in five healthy subjects datasets of the Human Connectome Project. The anatomical accuracy of the tracts was assessed by four judges with expertise in neuroanatomy, functional neurosurgery, and diffusion MRI. The variability among subjects was evaluated based on the fractional anisotropy and mean diffusivity of the tracts. Both multi-fiber approaches enabled the detection of complex fiber architecture in the basal ganglia. The qualitative evaluation by experts showed that the identified tracts were in agreement with the expected anatomy. Tract-derived measurements demonstrated relatively low variability among subjects. False-negative tracts demonstrated the current limitations of both methods for clinical decision-making. Multi-fiber tractography methods combined with state-of-the-art diffusion MRI data have the potential to help identify white matter tracts connecting DBS targets in functional neurosurgery intervention.

## Introduction

Deep brain stimulation (DBS) is an efficient neurosurgical treatment to alleviate motor symptoms, levodopa-induced motor fluctuations, and dyskinesia in Parkinson’s disease patients (Benabid et al., 1987; Krack and Hariz, 2013). The subthalamic nucleus (STN) and globus pallidus pars interna (GPi) have been established as effective targets (Wagle Shukla and Okun, 2014; Fasano and Lozano, 2015). Both nuclei are components of a large segregated cortical-subcortical network and DBS is likely to activate white matter fiber tracts connecting the targeted regions (Ashby et al., 1999; Gradinaru et al., 2009). As Parkinson’s disease is increasingly being viewed as a circuit disorder (DeLong and Wichmann, 2007; Lanciego et al., 2012), recent studies have investigated the changes in neural activity induced by DBS in the diseased circuits (McIntyre and Hahn, 2010). The white matter pathways that connect the STN and the globus pallidus (GP) include two sets of fibers: inhibitory pallido-subthalamic fibers that project from the globus pallidus pars externa (GPe) to the STN and excitatory subthalamo-pallidal fibers that project from the STN to the GPi and the GPe (Parent, 1995; Hamani et al., 2004). Tract tracing studies using invasive markers have been used in animal experiments to map the circuitry of these fibers (Shrink et al., 1996). In non-human primates, the pallido-subthalamic pathway presents with a complex topographical organization, with the pallidal input from the GPe extending over the entire rostrolateral two-thirds of the STN (Parent and Hzrati, 1995). Subthalamic neurons projecting to the GPe are predominantly located in the dorsolateral two-thirds of the STN whereas neurons projecting to the GPi are confined to the ventromedial third of the nucleus (Parent and Hzrati, 1995; Alkemade et al., 2015). Experimental studies in monkeys have demonstrated the subdivision of the basal ganglia into three functional motor, limbic and associative territories arising from the cerebral cortex (Haber et al., 1993; François et al., 1994; Karachi et al., 2005; Haynes and Haber, 2013). However, such studies cannot be reproduced in living humans. Magnetic resonance imaging (MRI) enables non-invasive exploration of the basal ganglia, and plays a critical role in the planning and execution of DBS (Dormont et al., 2010; De Celis Alonso et al., 2015). In the past decades, the introduction of diffusion MRI (dMRI) and subsequent development of tractography techniques have opened up the possibility to map the architecture of the brain white matter *in vivo* (Le Bihan et al., 1986; Basser et al., 1994; Pierpaoli and Basser, 1996; Mori et al., 1999). The analysis and interpretation of dMRI data using tractography tools have the potential to bring clinically relevant information for the planning and execution of DBS intervention (Henderson, 2012; Torres et al., 2014; Calabrese, 2016). In recent years, exploratory studies on the use of diffusion tensor imaging based on the single tensor model of diffusion for investigating white matter fibers connecting DBS targets have demonstrated promising findings (Barkhoudarian et al., 2010; Lemaire et al., 2011; Coenen et al., 2014; Rozanski et al., 2014; Sweet et al., 2014; Avecillas-Chasin et al., 2016; Vanegas-Arroyave et al., 2016). However, the tensor model can only identify a single fiber population within a voxel (Alexander et al., 2007). High angular resolution diffusion imaging (HARDI) acquisition sequences combined with sophisticated diffusion modeling approaches have been introduced to enable detection of multiple fiber orientations in complex neuroanatomical regions (Alexander, 2005). Such advanced techniques have been used for mapping white matter connections in the basal ganglia using dMRI data acquired on 7.0 Tesla scanners on healthy subjects and post-mortem specimens (Lenglet et al., 2012; Plantinga et al., 2014). While 7.0 Tesla scanners provide greater contrast than 3.0 Tesla scanners, such high-field MR scanners are not yet part of clinical routine. Through the Human Connectome Project (HCP) funded by the U.S. National Institutes of Health, cutting-edge neuroimaging data acquired on healthy young adults have been made publicly available to the scientific community. The HCP dMRI datatests include HARDI multi-shell data acquired on 3.0 Tesla scanners with customized coils and advanced dMRI acquisition sequences (David et al., 2013). Recent studies on HCP dMRI data have demonstrated the possibility to reconstruct the anatomical connectivity of complex pathways such as the orbitofrontothalamic fibers of passage using multi-fiber diffusion models (Makris et al., 2015). In this paper, we propose to investigate the connectivity between the subthalamic nucleus and the globus pallidus using two multi-fiber tractography approaches on five HCP subjects.

## Materials and Methods

### Anatomical Objective

Our study focused on the white matter anatomical connectivity between the STN and the GP. Fibers interconnecting these two regions consist of subthalamo-pallidal projections from the STN to the GPi and the GPe, and pallido-subthalamic projections from the GPe to the STN (Hamani et al., 2004). As these fibers cross the internal capsule in their trajectory, we included the tractography reconstruction of the pyramidal pathway.

### MRI Data Acquisition

We used the structural and dMRI datasets from five healthy subjects of the Human Connectome Project (**Table 1**). The data were acquired by the Washington University, University of Minnesota, and Oxford University (WU-Minn) HCP Consortium (Van Essen et al., 2013). All HCP subjects were scanned on the Connectome Skyra, which is a customized Siemens 3.0 Tesla Skyra scanner using a 32-channel head coil and a customized gradient set. The structural scans included T1-weighted and T2-weighted data that were acquired with the following parameters: T1-weighted: TE = 2.14 ms, TR = 2,400 ms, voxel size = 0.7 mm; T2-weighted: TE = 565 ms, TR = 3,200 ms, voxel size = 0.7 mm. The diffusion-weighted scans were acquired using a single-shot 2D spin-echo multiband Echo Planar Imaging (EPI) sequence. Each dMRI dataset consisted of 270 diffusion weighted images distributed equally over 3 shells defined with *b*-values of 1,000 s/mm^2^, 2,000 s/mm^2^, and 3,000 s/mm^2^, and nominal voxel size of 1.25 mm isotropic (Sotiropoulos et al., 2013; Uǧurbil et al., 2013). All five HCP diffusion-weighted imaging datasets used in this study had been pre-processed for intensity normalization, eddy-current, patient-motion and EPI distortion correction and co-registered to the anatomical scans (Glasser et al., 2013; Andersson and Sotiropoulos, 2015a,b).

**Table 1:** HCP subjects.

Subject	HCP Id	Age range	Sex
1	100307	26–30	Female
2	100408	31–35	Male
3	101915	31–35	Female
4	103414	22–25	Female
5	106016	31–35	Female

*The table presents the characteristics of the five healthy subjects of the Human Connectome Project that were used in the study.*

### MRI Data Post-processing

The tractography pipeline for the reconstruction of the targeted fascicles consisted of three steps: first, the segmentation of anatomical regions of interest, second the estimation of models based on the diffusion signal at each voxel, and third, the reconstruction and selection of the tracts.

#### Segmentation of Regions of Interest

**FIGURE 1:**
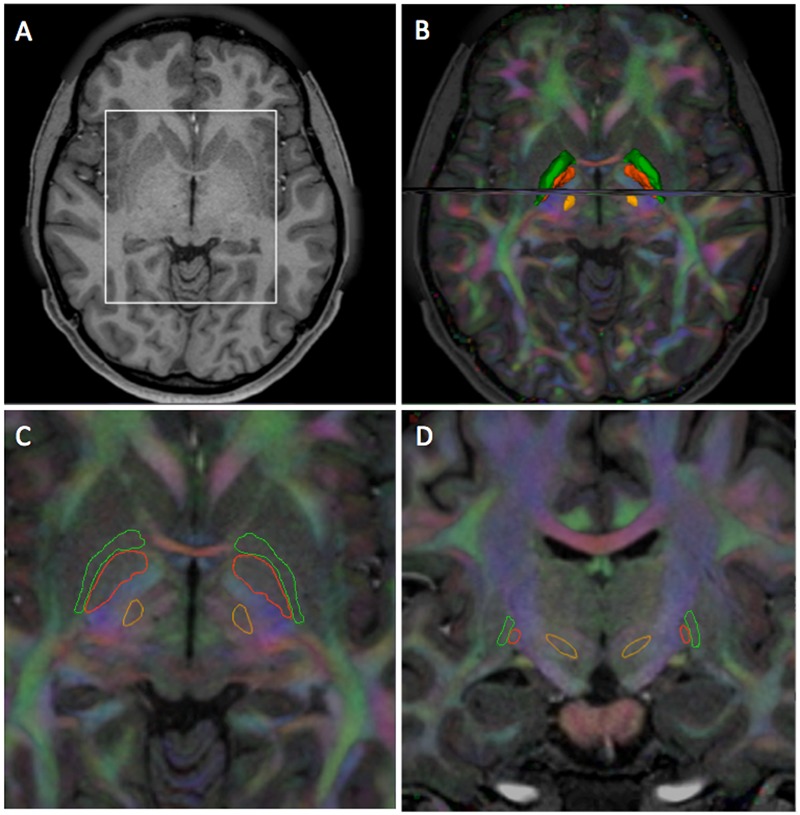
**Segmentation of the STN, GPi, and GPe in HCP subject 100307. (A)** Axial T1-weighted image. **(B)** 3D superior view of surface models of the STN (light orange), GPi (dark orange), GPe (green) with axial and coronal T1-weighted images overlaid on directionally encoded color (DEC) map. **(C)** Axial T1-weighted image overlaid on DEC map with outlined contours of the STN, GPi, and GPe. **(D)** Coronal T1-weighted image overlaid on DEC map with outlined contours of the STN, GPi, and GPe. The view presented in **(C)** corresponds to the ROI (white square) defined in **(A)**. The view presented in **(D)** corresponds to the coronal image displayed in **(B)**.

We defined two sets of anatomical regions-of-interest (ROIs) on the T1-weighted images for tractography purpose. A first set of ROIs for the reconstruction of the subthalamo-pallidal and pallido-subthalamic fibers was defined in the STN, GPe, and GPi. A second set of ROIs for the pyramidal tract was defined in the primary motor cortex, internal capsule and cerebral peduncles. Both sets of ROIs were generated within the 3D Slicer open-source platform for medical research (Fedorov et al., 2012). In the first set of ROIs, as the contours of the STN, GPe, and GPi were not directly visible in the T1-weighted images, we used an automated atlas-based segmentation approach implemented in the pyDBS software (D’Albis et al., 2015) (**Figure 1**). The method uses the Yeb Atlas, a unique 3D histological and deformable atlas of the basal ganglia that comprises 3D meshes of 80 structures identified on histological stainings from a post-mortem specimen (Yelnik et al., 2007). For each subject, 3D meshes of the STN, GPe, and GPi were generated from the T1-weighted images by deforming the Yeb Atlas using a global-to-local registration approach (Bardinet et al., 2009). The meshes were then voxelized in 3D Slicer to create ROIs with 0.3 mm voxel size for the tractography algorithms. In addition, the contours of the GPe and GPi were manually edited to integrate the anatomical information contained in the dMRI data. The meshes of the edited structures were then re-generated from the edited labelmaps using the Marching Cubes algorithm (Lorensen and Cline, 1987). In the second set of ROIs, the primary motor cortex was annotated by positioning fiducials in the pre-central gyrus of a volume-rendered image of the T1-weighted scan. The cerebral peduncles and posterior limb of the internal capsule were manually segmented in axial cross-sections of the directionally encoded color map derived from the diffusion-weighted images in 3D Slicer.

#### Multi-Fiber Models

We used two different multi-fiber approaches to estimate the orientation of white matter fibers from the diffusion-weighted signal arising at each voxel: a multi-compartment (MC) model and a multi-shell multi-tissue constrained spherical deconvolution (MSMT-CSD) method.

**FIGURE 2:**
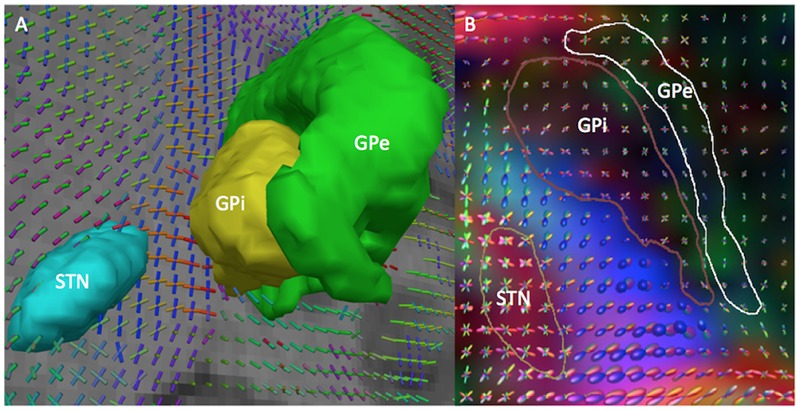
**3D visualization of the principal directions of diffusion computed by the MC and MSMT-CSD diffusion models in HCP subject 100307. (A)** MC model. The figure shows a 3D coronal view of the ball-and-stick glyphs representing the two principal directions of diffusion at each voxel. The glyphs are overlaid on a coronal T1-weighted image with 3D models of the STN (cyan), GPi (yellow), and GPe (green). **(B)** MSMT-CSD model. The figure shows a 3D superior view of the spherical harmonics glyphs representing the principal directions of diffusion. The glyphs are overlaid on a DEC map with contours of the STN (yellow), GPi (dark brown), and GPe (white).

The MC approach uses the ball-and-stick model to represent the diffusion-weighted signal as arising from a sum of multiple anisotropic components, a single isotropic diffusion compartment and volume fractions thereof (Behrens et al., 2007). We fitted the ball-and-stick model to the dMRI data using a using a Bayesian estimation procedure implemented in FSL XFibres (Jenkinson et al., 2012). Parameter settings included the continuous exponential approach for multi-shell data and a maximum of three fiber compartments per voxel. **Figure 2A** shows an example of 3D visualization of the principal directions of diffusion at each voxel identified using the MC method.

The MSMT-CSD method uses a continuous distribution of fiber orientations at each voxel. In this approach, the diffusion-weighted signal arising from different fibers population is represented as an integral over the distribution of fiber orientations, and therefore can be expressed as the spherical convolution of the fiber Orientation Distribution Function (fODF) and the diffusion-weighted signal profile from a single fiber orientation (Tournier et al., 2004). We estimated the fODF at each voxel with a maximum of eight spherical harmonics using the MSMT-CSD algorithm implemented in the Mrtrix software (Jeurissen et al., 2014). **Figure 2B** shows an example of 3D visualization of the principal directions of diffusion at each voxel identified using the MSMT-CSD approach.

#### Fiber Tracking Algorithms

For the MC approach, deterministic tractography was performed using a fiber tracking method similar to the eXTended multi-fiber streamline tractography approach (Qazi et al., 2009), which consisted of a custom implementation used in combination with a model-based estimation framework for interpolation (Cabeen et al., 2013). The fiber tracking algorithm used a step size of 0.5 mm and 25 seeds per voxel, which were placed in a one-voxel neighborhood surrounding each ROI. The ball-and-stick diffusion models were interpolated using a data-adaptative kernel regression framework (Cabeen et al., 2013, 2016). The framework used clustering-based optimization and two parameters: spatial bandwidth, which controls the smoothness of the interpolation, and model selection, which controls the number of estimated compartments. In this study, we used a spatial bandwidth of 1.0 mm and model selection parameter *λ* = 0.9999, corresponding to up to three fiber compartments. Tracking was terminated upon reaching a target ROI, when the angle changed more than 55°, or when a compartment’s volume fraction dropped below 0.05.

For the MSMT-CSD approach, whole brain probabilistic anatomically constrained tractography (Smith et al., 2012) was performed with the following parameters: 10 millions tracts, step length, 0.625 mm; curvature threshold, 45°; minimum tract length, 10 mm; maximum tract length, 250 mm; and fiber orientation distribution amplitude cut-off, 0.1. The spherical-deconvolution informed filtering of tractograms (SIFT) was applied to reduce the number of streamlines to five millions to provide a biologically meaningful estimate of structural connection density by removing false positive tracts (Smith et al., 2013). The tracts of interest in the resulting whole-brain tractogram were isolated using MrTrix.

In the rest of the paper, we designate the MC deterministic tractography approach as MC-det and the MSMT-CSD probabilistic approach as CSD-prob.

#### Tract Selection Strategy

The following section describes the tract selection strategy chosen for the MC-det and CSD-prob methods. In the MC-det approach, tractography seeds for the STN-GPi pathway were placed in one voxel shells surrounding both STN and GPi ROIs, and tracks were terminated with reaching either ROI. For the STN-GPe pathway, tractography seeds were placed in one voxel shells surrounding both STN and GPe ROIs, tracks were allowed to pass through the GPi and were terminated with reaching either the STN or the GPe ROI. For the pyramidal tract, tractography was seeded in three ROIs: white matter neighboring the precentral gyrus, the posterior limb of the internal capsule, and cerebral peduncle. Pyramidal fibers were retained only if they intersected all three ROIs and did not intersect either the GPi or GPe ROIs. The reconstructed tracts were visualized as streamlines with 3D Slicer.

In the CSD-prob approach, after whole brain tractography reconstruction, fibers passing through the STN, GPi, and GPe were selected for STN-GPi pathway, and fibers passing through the STN and GPe were selected for the STN-GPe pathway. Fibers passing through the motor cortex, the posterior limb of the internal capsule and the cerebral peduncles were selected for the pyramidal tract. Tract density maps (TDMs) were then calculated for all reconstructed pathways (Calamante et al., 2010), and the tracts were visualized as volume-rendered TDM images using the GPU-based volume rendering functionality of 3D Slicer.

### Evaluation of the Tractography Reconstructions

#### Evaluation by Anatomical Experts

The qualitative evaluation of the anatomical accuracy of the tracts was performed by four experts in human neuroanatomy that include two neuroanatomists, a neurosurgeon and a neuroradiologist. For each subject, subthalamo-pallidal, pallido-subthalamic, and pyramidal tracts reconstructed using the MC-det and the CSD-prob methods were loaded into the 3D Slicer platform, along with the 3D models of the subthalamic nucleus and globus pallidus. The tracts and 3D models were overlaid on T1-weighted images, T2-weighted images and directionally encoded color maps. The experts reviewed the reconstructed white matter tracts using the interactive 3D visualization functionalities of the software. Each reviewer could adjust the transparency of the tracts and models, select which tract to view and which to hide, zoom in and out, and rotate the tracts to best demonstrate anatomical relationships between the different structures. The evaluation of the tracts was performed using four criteria: the topographical localization of each tract; the starting and ending regions of the fascicles; the specific shape of the fascicles, e.g., fanning shape of the pyramidal pathway from the cortex to the internal capsule; the relationships between the different bundles, e.g., comb organization of the subthalamic fasciculus and pyramidal tract. These criteria were used to evaluate the anatomical accuracy of the tracts based on the similarity between the tractography reconstructions and known neuroanatomy. The experts assigned a score ranging from 5 (excellent) to 1 (poor) averaged on the criteria used for the review of each fascicle.

#### Tract-Derived Measurements

In order to assess the variability among subjects, we calculated the fractional anisotropy (FA) and mean diffusivity (MD) of the envelope of the tracts reconstructed by both methods. For the MC-det method, the envelopes of the tracts were generated by converting the streamlines into voxelwise binary labelmaps, with label = 1 when a tract had been detected and label = 0 when no tract was detected. For the CSD-prob method, the TDM volumes were thresholded to remove voxels with probability values lower than 0.05 and the resulting maps were converted into binary labelmaps. For each subject, we calculated the FA and MD volumes from the dMRI data using the 3D Slicer software, and we computed the mean and standard deviation of the FA and MD values inside the envelopes of the MCM-det and CSD-prob tracts.

### Statistical Analysis

We performed a statistical analysis of the scores given by the judges and the tract-derived measurements. For each tract, we calculated the mean and standard deviation of the average scores given by the four judges for the MC-det and CSD-prob methods to estimate the variability in the anatomical accuracy of the reconstructed tracts across subjects. In order to assess the level of agreement among the four judges, we computed the intraclass correlation coefficient (ICC) of the scores for each tractography method. In addition, we characterized the distribution of FA and MD values for each tract using box plots to evaluate the variability of tract-derived metrics among subjects.

## Results

**FIGURE 3:**
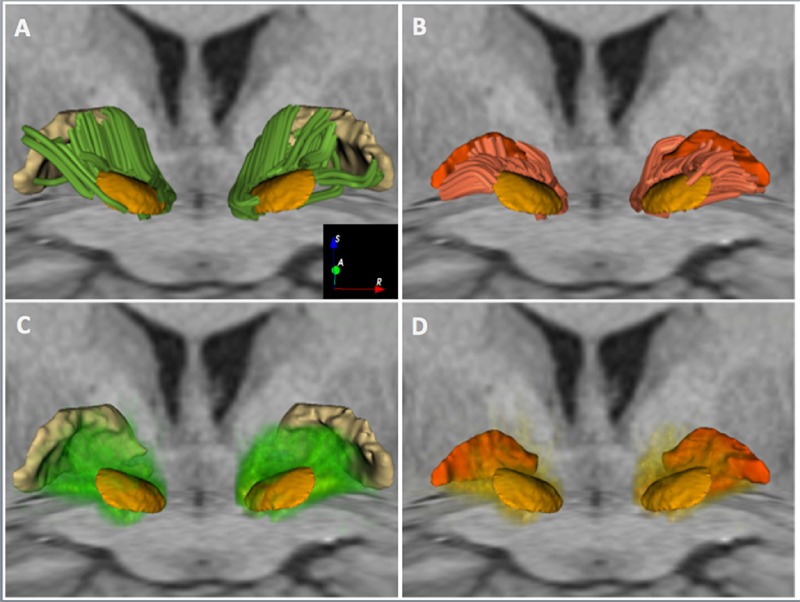
**White matter connectivity between the STN and the GP in HCP subject 100307.** The figure shows an anterior 3D view of MC-det tracts represented as streamlines (top row) and CSD-prob tracts represented as volume-rendered tract density maps (bottom row). The tracts are superimposed on an axial T1-weighted image at the level of the cerebral peduncles and a coronal T1-weighted image at the level of the anterior commissure, along with 3D surface models of the STN (light orange), GPi (dark orange), and GPe (light yellow). **(A)** and **(C)** show tracts interconnecting the STN and the GPe. **(B)** and **(D)** show tracts interconnecting the STN and the GPi.

**FIGURE 4:**
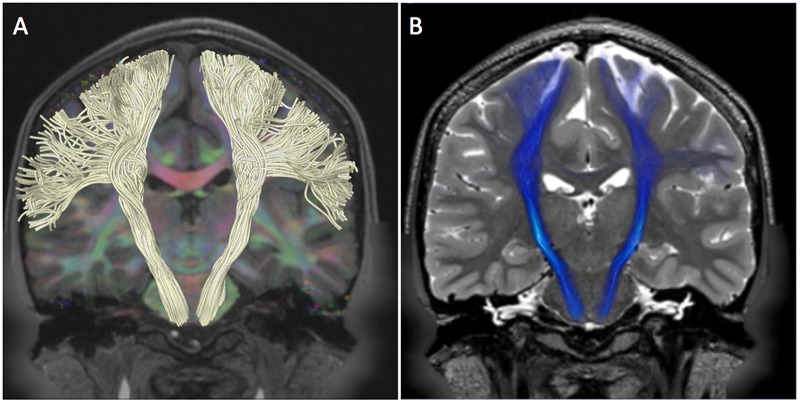
**Pyramidal pathway in HCP subject 100307.** The figure shows the tractography reconstruction of pyramidal white matter fibers arising from the pre-central gyrus. **(A)** Pyramidal pathway (white) reconstructed using the MC-det approach. The tracts are displayed on a DEC map overlaid on a coronal T1-weighted image. **(B)** Pyramidal pathway (blue) reconstructed using the CSD-prob approach. The tracts are displayed on a coronal T2-weighted image.

**FIGURE 5:**
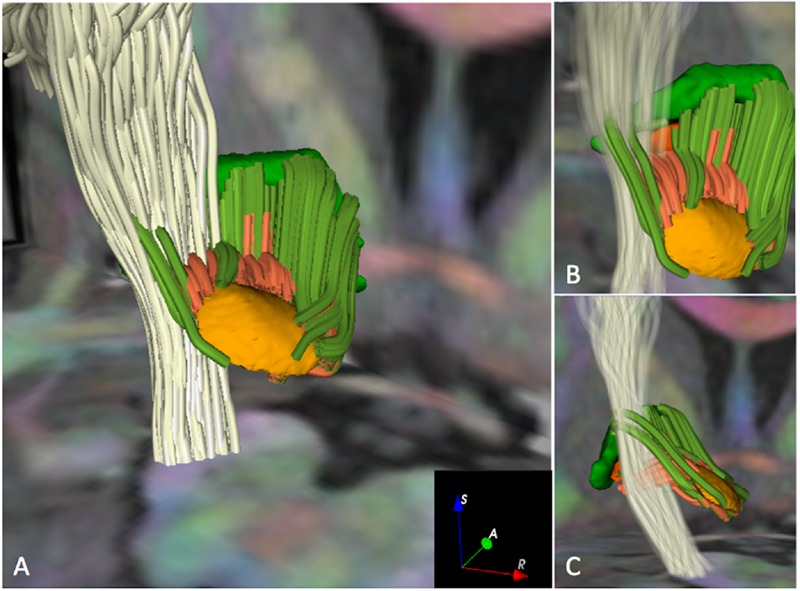
**Crossings of the pyramidal pathway with subthalamo-pallidal and pallido-subthalamic fibers (MC-det approach) in HCP subject 100307.** The figure shows 3D postero-superior views of the intersection of the pyramidal pathway (white streamlines) with fibers interconnecting the STN and the GPi (dark orange streamlines) and fibers interconnecting the STN and the GPe (dark green streamlines). An axial T1-weighted image at the level of the cerebral peduncles and a coronal T1-weighted image at the level of the anterior commissure with 3D surface models of the STN (light orange), GPi (dark orange), and GPe (green) are displayed for anatomical reference. A DEC map is overlaid on the T1-weighted images. The opacity and number of pyramidal fibers displayed in **(A)** have been reduced in **(B)** and **(C)** to enable the visualization of the crossings of the motor pathway with fibers interconnecting the STN with the GPi and GPe.

**FIGURE 6:**
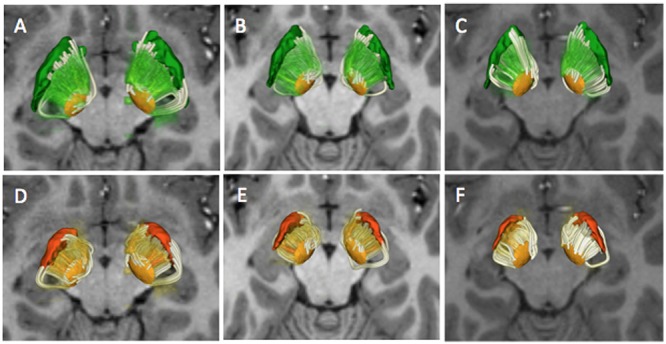
**Combined deterministic and probabilistic tractography reconstructions.** The figure shows a superior 3D view of the deterministic and probabilistic tracts interconnecting the STN and the GPe (top row) and STN and GPi (bottom row) in three subjects (**A–D**: HCP 100408; **B–E**: HCP:101915; **C–F**: HCP 103414). The MC-det tracts are represented as streamlines (white) and the CSD-prob tracts are represented as volume-rendered imaged (green, orange). The tracts are superimposed on an axial T1-weighted image at the level of the cerebral peduncles with 3D surface models of the STN (light orange), GPi (dark orange), and GPe (green).

**FIGURE 7:**
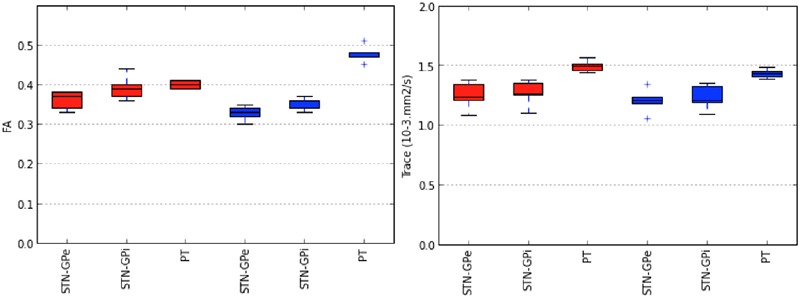
**Variability in tract-derived measurements.** The figure presents box plots of fractional anisotropy (FA) and mean diffusivity (MD) of tracts interconnecting the STN and the GPe (STN-GPe), tracts interconnecting the STN and the GPi (STN-GPi) and pyramidal tract (PT). The lines in each box correspond to the median, interquartile range, minimum and maximum values of the tract-derived measurements. Tracts identified by the MCM-det method are represented in red. Tracts identified by the CSD-prob methods are represented in blue.

The subthalamo-pallidal, pallido-subthalamic, and pyramidal tract were successfully identified on all five subjects using the MC-det and CSD-prob methods. **Figures 3**, **4**, and **5** show individual tractography reconstructions of the three pathways in a single subject. **Figure 5** shows the crossings of the pyramidal tract with subthalamo-pallidal and pallido-subthalamic tracts. **Figure 6** shows combined MC-det and CSD-prob tractography reconstruction of the subthalamo-pallidal and pallido-subthalamic tracts in three subjects, and illustrates the agreement between methods. Overall, the neuroanatomists rated the reconstructed tracts as consistent with the expected anatomy. **Table 2** presents the results of the qualitative evaluation of the tracts. The average and standard deviation of the scores for the STN-GPe, STN-GPi, and PT were 3.3 ± 0.2, 3.7 ± 0.3, and 3.5 ± 0.2, respectively. In addition, the calculation of the ICC showed a good agreement among judges for both tractography methods: the ICC for the MCM-Det method was 0.70 with 95% confidence interval of (0.39–0.97) and the ICC for the CSD-det methods was 0.79 with 95% confidence interval of (0.51–0.98). **Figure 7** shows the box plots of the tract-derived measurements computed for each tract and each method. Results show a relatively low variability of FA and MD values among subjects, and similar distributions of FA and MD values between methods.

**Table 2:** Summary of the qualitative evaluation of the reconstructed tracts.

Tract	Subject 1	Subject 2	Subject 3	Subject 4	Subject 5	Average (*SD*)
STN-GPe	3.6	3.3	3.5	3.1	3	3.3 (0.2)
STN-GPi	4.1	3.5	3.5	3.6	3.9	3.7 (0.3)
PT	3.8	3.2	3.5	3.5	3.4	3.5 (0.2)

*The table presents the average review score of the tracts interconnecting the STN and the GPe (STN-GPe), tracts interconnecting the STN and the GPi (STN-GPi), and the pyramidal tract (PT) for each subject. The scores were given using a scale of 1–5 (1 – poor, 2 – fair, 3 – good, 4 – very good, and 5 – excellent).*

### STN-GPE Connections

In both the MC-det and CSD-prob methods, the majority of fibers interconnecting the STN and the GPe crossed the anterior two-thirds of the posterior limb of the internal capsule. This finding is in agreement with the presence of the descending pyramidal tract in the remaining third of the posterior limb. Most fibers reconstructed using the MC-det method emerged from the mediodorsal part of the GPe, while the central and ventral parts presented less fibers (**Figure 3A**). Fibers reconstructed using the CSD-prob connected the STN to both the mediodorsal and medioventral aspects of the GPe (**Figure 3C**). The method did not identify fibers on the upper most part of the mediodorsal aspect of the GPe.

### STN-GPi Connections

Both the MC-det and CSD-prob methods identified tracts connecting the STN to the GPi. Most of the tracts arose from the ventromedial aspect of the STN (**Figures 3B,D**). The MC-det method detected a smaller number of tracts arising from the mediodorsal aspect of the STN (**Figure 3B**) compared to the CSD-prob method (**Figure 3D**).

### Pyramidal Tracts

The pyramidal pathway was correctly depicted by the MC-det method (**Figure 4A**) and CSD-prob method (**Figure 4B**) at the level of the posterior limb of the internal capsule and cerebral peduncles. Lateral projections of the pyramidal pathway arising from the hand, face, lips and tongue area were identified by both the MC-det (**Figure 4A**) and the CSD-prob approach (**Figure 4B**). The CSD-prob approach identified a relatively smaller number of projections to most lateral lips and tongue area, and included false positive tracts belonging to the corpus callosum (**Figure 4B**). The streamline visualization of MC-det reconstructed tracts enabled three-dimensional depiction of the crossings of subthalamo-pallidal and pallido-subthalamic fibers with the descending pyramidal tract at the level of the internal capsule (**Figure 5**). Fibers crossings showed the comb fascicles organization (**Figures 5B,C**) (Nauta and Mehler, 1966).

## Discussion

This paper presents an exploratory study on the white matter connectivity between the subthalamic nucleus and the globus pallidus on healthy subjects data from the Human Connectome Project. We have shown that using multi-fiber modeling approaches and high-resolution multi-shell dMRI data at 3.0 Tesla, it is possible to detect complex fiber architecture in the basal ganglia. We chose to analyze the data using two different multi-fiber methods in order to confirm that our findings were not a method-related artifact. The results show that both approaches enabled the reconstruction of subthalamo-pallidal and pallido-subthalamic fibers. The review by four neuroanatomy experts demonstrated that overall the reconstructed tracts where in agreement with known anatomy. The main advantage of the MC-det and CSD-prob methods arises from the use of advanced multi-fiber models which enable the identification of complex anatomical configurations in voxels where multiple fiber population cross, such as at the intersection of the descending pyramidal tract with the pallido-subthalamic and subthalamo-pallidal fibers. However, both approaches also present some disadvantages. The MCM-det approach relies on the ball-and-stick model that is limited in the detection of fanning tracts. The volume rendered images of the TDMs generated by the CSD-prob methods can be difficult to interpret. In addition, both deterministic and probabilistic tractography methods propagate streamlines by considering only directional information at the voxel level and ambiguities can occur in complex anatomical regions.

While we used two advanced multi-fiber models and high-quality data, the tractography results still present some limitations. First, the majority of pallido-subthalamic and subthalamo-pallidal fibers identified by the MC-det approach were located on the ventral aspect of the STN. **(Figures 3A,B**), and the streamlines seem to stop just before entering the nucleus (**Figure 5**). A potential explanation of the absence of tracts on the dorsal aspect of the STN can be given by the presence of the H2 field of Forel where the lenticular fasciculus and the ansa lenticular merge to form a dense bundle of fibers (Dejerine, 1901). The lenticular fasciculus projects from the GPi, crosses the internal capsule, reaches the H2 and H fields of Forel, and courses superiorly and anteriorly through H1 field of Forel to enter the thalamus. The ansa lenticularis projects infero-medially from the GPi, crosses the internal capsule, travels towards the STN and turns superiorly to pass through H and H1 before entering the thalamus. As the focus of our study was to investigate the connectivity between the STN and the GP, we did not include the tractography reconstruction of these two pathways. However, the anatomical complexity of the ansa lenticularis and the lenticular fasciculus and their close proximity to the pallido-subthalamic and subthalamo-pallidal fibers might have affected the anatomical accuracy of the tractography results and introduced false-negative tracts. The apparent distance between the extremities of the streamlines and the nuclei visible in **Figure 5** is likely due to the generation of the tractography ROIs. The ROIs were voxelized from the 3D meshes of the nuclei, which had been generated from a high-resolution histological atlas with 0.16 mm × 0.16 mm × 0.41 mm voxel size. During the voxelization process, we used as a reference the T1-weigthed HCP scan resampled to 0.3 mm. The difference in voxel size between the original mesh and the voxelized ROI likely introduced a few tenth of millimeters difference that may explain the visual gap between the extremities of the tracts and the nuclei.

Second, we observed a discrepancy between the two methods in the tractography reconstruction of fibers interconnecting the STN and the GPe. Tracts generated using the MC-det approach were mostly located on the upper dorsal part of the GPe while tracts generated using the CSD-prob approach were located in the ventrolateral and mediolateral part of the GPe. The MC-det approach used a ball-and-stick model for the detection of crossings fibers. However, the model presents limitations for the detection of fanning fibers, which may be encountered within the globus pallidus (Sotiropoulos et al., 2012). In addition, the relatively smaller number of fibers detected by both methods may be due to the presence of the striato-pallido-nigral pathway crossing the pallidum. Striato-pallido-nigral fibers arise from the striatum, travel through the GPe and the GPi and cross the internal capsule to reach the substantia nigra (Percheron et al., 1984). Pallido-subthalamic and subthalamo-pallidal fibers within the GPe and GPi are likely to be less dense that striato-pallido-nigral fibers, which might explain the smaller number of fibers detected (Percheron et al., 1984). In addition, the CSD-prob approach included in the descending pyramidal pathway false positive tracts belonging to the corpus callosum. This limitation is likely due to the high number of kissing fibers along the trajectory of the pyramidal tract, and its close location to the corpus callosum.

Finally, despite the high-resolution of the HCP dMRI data, the difference in scale between the microscopic size of myelinated axons and the macroscopic 1.25 mm voxel size of the diffusion-weighted images remains significant. As a consequence, each voxel is likely to contain multiple axons belonging to several different pathways. Further studies are needed to investigate the complex spatial relationship between the pallido-subthalamic and subthalamo-pallidal fibers with the ansa lenticularis, lenticular fasciculus and striato-pallido-nigral pathway. Future work will include the tractography reconstruction of these complex pathways as well as their crossings with the internal capsule, and will explore the use of a higher number of compartments in the MC-det method and a larger number of harmonics of the CSD-prob methods (Tournier et al., 2012).

While the continuous refinement of mathematical models of diffusion and fiber tracking techniques enables the non-invasive exploration of the connectivity of the human white matter, dMRI tractography remains a research tool. Studies have demonstrated that dMRI tractography presents limitations for neurosurgical decision-making in the resection of brain gliomas (Pujol et al., 2015). The false-negative findings of our exploratory work demonstrate that tractography cannot currently replace electrophysiology mapping or intra-operative MR imaging in functional neurosurgery. However, as the technology continues to progress, dMRI tractography has the potential to become part of the apparatus of brain mapping tools to help understand the clinical effects of DBS interventions. The HCP datasets used in this study have been acquired with optimized multiband pulse sequences on a customized Siemens 3T Connectome scanner and pre-processed for noise removal and artifacts minimization. The HCP dMRI scans represent the state-of-the-art dMRI data currently available in brain research, and are expected to represent the clinical dMRI scans that will be available in neurosurgery departments in the upcoming years. Our preliminary study aimed at exploring the tractography reconstructions that could become available to assist with pre-surgical planning of DBS intervention in neurosurgery clinics in the near future.

## Conclusion

We have presented a pilot study on the connectivity between the subthalamic nucleus and the globus pallidus using two multi-fiber tractography approaches on dMRI data acquired on five healthy subjects of the Human Connectome Project. Our analysis revealed that multi-fiber tractography enables three-dimensional visualization of complex neuroanatomical fibers in the basal ganglia. However, false-negative tracts have demonstrated the current limitations of the techniques. As progresses are continuously being made in the development of advanced mathematical models of diffusion and fiber tracking algorithms, dMRI tractography has the potential to become a useful component of the brain mapping apparatus available to clinicians for functional neurosurgery intervention.

## Author Contributions

Substantial contributions to the conception or design of the work: SP, EB, JY, CF, PJ, and RK. Substantial contributions to the data analysis: SP, RC, SBS, SFV, YZ, EB, and RK. Substantial contributions to the interpretation of data with anatomical expertise: SP, JY, CF, GRC, and RK. Substantial contributions to drafting the work: SP, RC, SBS, RK, and EB. Substantial contributions to revising the work: SP, RC, SBS, JY, CF, SFV, CK, YZ, GRC, PJ, RK, and EB. Agreement to be accountable for all aspects of the work in ensuring that questions related to the accuracy or integrity of any part of the work are appropriately investigated and resolved: all authors SP, RC, SBS, JY, CF, SFV, CK, YZ, GRC, PJ, RK, and EB.

## Conflict of Interest Statement

The authors declare that the research was conducted in the absence of any commercial or financial relationships that could be construed as a potential conflict of interest.

## Abbreviations

DBS: deep brain stimulation
dMRI: diffusion MRI
GP: globus pallidus
GPe: globus pallidus pars externa
GPi: globus pallidus pars interna
HCP: Human Connectome Project
MC: multi-compartment
MSMT-CSD: multi-shell multi-tissue constrained spherical deconvolution
PD: Parkinson’s disease
ROI: region of interest
STN: subthalamic nucleus
